# Isolation and Characterization of 13 New Polymorphic Microsatellite Markers in the *Phaseolus vulgaris* L. (Common Bean) Genome

**DOI:** 10.3390/ijms130911188

**Published:** 2012-09-07

**Authors:** Aihua Wang, Yi Ding, Zhenhua Hu, Chufa Lin, Shuzhen Wang, Bingcai Wang, Hongyuan Zhang, Guolin Zhou

**Affiliations:** 1 Hubei Provincial Engineering Research Center of Vegetable Seeds and Seedlings, Wuhan Vegetable Research Institute, Wuhan Academy of Agricultural Science & Technology, Wuhan 430345, China; E-Mails: bigli_78@163.com (A.W.); 13035103237@163.com (Z.H.); cflin3228@sina.com (C.L.); 1337009140@163.com (B.W.); 2 State Key Laboratory of Hybrid Rice, Department of Genetics, College of Life Sciences, Wuhan University, Wuhan 430072, China; E-Mails: yiding@whu.edu.cn (Y.D.); wangshuzhen04@163.com (S.W.); ilove9112@126.com@126.com (H.Z.)

**Keywords:** microsatellite markers, *Phaseolus vulgaris* L., cross-species transferability, genetic diversity

## Abstract

In this study, 13 polymorphic microsatellite markers were isolated from the *Phaseolus vulgaris* L. (common bean) by using the Fast Isolation by AFLP of Sequence COntaining Repeats (FIASCO) protocol. These markers revealed two to seven alleles, with an average of 3.64 alleles per locus. The polymorphic information content (PIC) values ranged from 0.055 to 0.721 over 13 loci, with a mean value of 0.492, and 7 loci having PIC greater than 0.5. The expected heterozygosity (*H*_E_) and observed heterozygosity (*H*_O_) levels ranged from 0.057 to 0.814 and from 0.026 to 0.531, respectively. Cross-species amplification of the 13 prime pairs was performed in its related specie of *Vigna unguiculata* L. Seven out of all these markers showed cross-species transferability. These markers will be useful for future genetic diversity and population genetics studies for this agricultural specie and its related species.

## 1. Introduction

*Phaseolus vulgaris* L. (common bean, 2*X* = 2*n* = 22) belonging to the Fabacaea family, is a self-pollinated vegetable and the most important legume for human consumption in the world, widely cultivated in North America, South America and Africa. Therefore, it is important to study the genetic diversity, population ecology, evolution and conservation of this species using molecular tools, such as microsatellite markers.

Until now, EST-SSR markers [1,2], BAC-derived microsatellite [3] and microsatellite markers based on non-enriched, small-insert genomic libraries [4] of the common bean have been developed to study the genetic diversity of the common bean population. However, EST-derived SSRs (Simple Sequence Repeats) rely on sets of expressed sequence tags (ESTs) in the public databases and can only be obtained from sequences that are expressed. In addition, BAC-derived microsatellite and microsatellite markers based on non-enriched, small-insert genomic libraries are dependent on the libraries in the public databases. In contrast, the Fast Isolation by AFLP of Sequences Containing repeats (FIASCO) method can quickly identify SSRs throughout the entire genome without knowing the genomic information of a given species. In addition, genomic SSR markers are more polymorphic than EST-SSRs. In this paper, we developed 13 microsatellites for *P. vulgaris* using the FIASCO method.

## 2. Results and Discussion

A total of 120 positive colonies with the fragment of expected size were sequenced. Within them, 55 unique microsatellites with sufficient flanking regions were chosen to design prime pairs. All of the 55 primer pairs were surveyed in 38 individuals of *P. vulgaris* provided by the Wuhan Vegetable Research Institute, Wuhan Academy of Agricultural Science & Technology, and collected from different areas in China. Thirteen primer pairs were identified as polymorphic, with a variable number of alleles per locus. In this study, we developed 13 effectives primers from 120 positive colonies and the effectives primer rate was 10.8% (13/120), which is a higher primer rate than the results recently reported by Blair *et al.* (2011) of 5.9% (184/3123) [2] and by Garcia *et al.* (2011) of 3.2% (302/9583) derived from EST-SSR [5], respectively. The effectives primer rate in our results is also higher than the results reported by Córdoba *et al.* (2010), in which the effectives primer rate is only 0.64% (623/98017) from the BAC-derived SSRs [3], and the results reported by Blair *et al.* (2009) based on screening of non-enriched, small-insert genomic libraries (microsatellite frequency overall appears to be less than 1% of clones) [4]. It is likely that the effective primer rate differences between various results reported by different researchers are due to the source sequences differences (coding or non-coding regions of the genome) used, or the different methods of generating the SSR library, or the different methods of identification the microsatellites, or the specific species studied. In this study, we use the FIASCO method to identify SSRs throughout the entire genome, apparently, the effectives primer rate was higher than that of other methods, this shows that our method used in this research is superior to other methods. In our results, the average number of alleles per locus was 3.64, and the allele number ranged from two to seven (Table 1). The polymorphic information content (PIC) values ranged from 0.055 to 0.721 over the 13 loci and 7 loci had a PIC greater than 0.5, with a mean value of 0.492, which was higher than that of the EST-derived SSR markers [2,5]. This is in agreement with the results of Garcia *et al.* [5]. The expected heterozygosity (*H*_E_) and observed heterozygosity (*H*_O_) levels ranged from 0.057 to 0.814 and from 0.026 to 0.531, with a mean values of 0.453 and 0.165 respectively (Table 1), which was similar to a previous study revealed by EST-SSRs [6].

The exact tests for Hardy–Weinberg equilibrium (HWE) revealed that 11 of the 13 microsatellites showed significant deviation from HWE (*p* < 0.05) [7] (Table 1). The reason for this may be that *P. vulgaris* is a predominantly self-pollinated species, which results in a low rate of random mating of different common bean species. Furthermore, the polymorphic estimating of these primers developed in a cultivation of small groups of 38 samples. No significant linkage disequilibrium (LD) was found between these polymorphic loci following the Bonferroni correction [8], which demonstrated that the pairs of loci developed in our study were distributed on different chromosomes. In addition, cross-species amplification of the 13 primer pairs was performed in the related species *Vigna unguiculata* L. The frequencies of null alleles for those loci that showed significant deviation from HWE (*p* < 0.05) were tested using the micro-checker software [7]. Of these primers, seven (C136, C130, C115, C132, C106, C45, and G10) could be successfully transferred to the tested species (Table 1) and the rate of transferability was 53.85%, but Garcia *et al*. (2011) reported a higher rate of transferability of 93.8% of the EST-SSRs [5]. The higher transferability rate of EST-SSRs can be explained by their correspondence to the transcribed component of a gene unit, which confers a high potential for inter-specific transferability [9]. After sequencing confirmation of the SSR-containing regions and the flanking sequences of the amplified bands, the microsatellite loci of *V. unguiculata* detected by the seven primer pairs were consistent with those detected in *P. vulgaris*.

## 3. Experimental Section

The genomic DNA of *P. vulgaris* was extracted from seeds using the CTAB (cetyltrimethyl ammonium bromide) method [10] with slight modifications. An (AG)*_n_*-enriched partial genomic library was then constructed following the FIASCO protocol with certain modifications. Briefly, total genomic DNA was digested with the restriction enzyme *Mse*I (New England BioLabs). After ligating *Mse*I adapters to the digested DNA, amplification was performed using an *Mse*I primer. The amplified DNA fragments were denatured and hybridized to a 5′-biotinylated-(AG)*_n_* probe. The enriched fragments containing microsatellite repeats were ligated into a pGEM-T vector (Promega, Madison, USA) and transformed into *Escherichia coli* TOP10 competent cells. The transformants were cultured, and positive colonies were selected and PCR-tested to determine the insert size. A total of 140 positive colonies with a fragment of the expected size were sequenced using a HiSeq 2000 DNA sequencer (Illumina, University of Utah, USA). These sequences were analyzed for repeat motif regions of microsatellites using the software TANDEM REPEATS FINDER [11]. The Primer Premier 5 software [12] was used to design primer pairs for these regions.

The PCR conditions were optimized for each selected primer pair. All of the amplification reactions were performed in a single block of a PX2 ThermalCycler (Thermo Hybaid). The PCR amplifications were performed in a volume of 15 μL containing 20 ng genomic DNA, 2.5 μL 10× PCR buffer with MgCl_2_, 1 μM each primer, 250 μM each dNTP and 1 U *Taq* DNA polymerase (Biocolors). The following thermal profile was used for the PCR amplifications: initial denaturation at 95 °C for 5 min, followed by 30 cycles of 94 °C for 40 s, annealing at the optimal temperature (Table 1) for 45 s, and 72 °C for 40 s, and a final 10 min elongation step at 72 °C. The fragments were separated on 8% (*w/v*) denaturing polyacrylamide sequencing gels and visualized by silver nitrate staining. The size of the amplified fragments was determined by comparison with the pBR322 DNA/*Msp*I DNA marker of 26–622 bp (Tiangen, Beijing, China).

Polymorphisms of all of the primer pairs were then surveyed in 38 individuals. The SSR band for each was treated as a locus, and a matrix for the SSR phenotypes was assembled. The matrix was used for the statistical analyses below. The data were calculated using the ARLEQUIN 3.01 software [13]. Several parameters of genetic diversity, including the *H*_E_, *H*_O_, number of alleles (*N_a_*) per locus, LD, HWE, and PIC, were calculated using Cervus version 3.0.3.

## 4. Conclusions

The FIASCO method, which quickly identifies SSRs throughout an entire genome, has previously been applied in the sacred lotus [14,15]. In the current study, we first confirmed that this method can also be used in the common bean and that cross-species amplification with the identified markers can be performed in the related species of cowpea. Hence, the polymorphic microsatellite markers developed in this study will be useful in the construction of genetic linkage maps, classification of germplasm and identification in the breeding of *P. vulgaris*, as well as in the study of the phylogeny, origin and evolution of *Leguminosae* crops.

## Figures and Tables

**Table 1:** Characteristics of the 13 microsatellite primers developed in *P. vulgaris*.

Locus	Primer Sequence (5′*–*3′)	Repeat Motif	Size Range (bp)	*H*o	*H*e	*N*_a_	D	PIC	*T*_a_ (°C)	GenBank
C9	F:ACAGAGACGAGTGCGTGAGAGTTAG	(AT)_15_	445–470	0.469	0.428	4	*	0.471	57	JQ739888
	R:AAAGACAGTTCTAGGAAGAACCGTC									
C33	F: CTCTTTCTGCTTCCTTTCTACGC	(AG)_15_	536–565	0.531	0.814	7	*	0.721	59	JQ739889
	R:TTCTTCACAGTCAAGGGAGTAGAAG									
C42	F:TGTCATAAACCTGCTGGTGAATAAC	(AG)_28_	287–342	0.026	0.473	3	NS	0.444	60	JQ739900
	R:CCTATTTCAGAATCACAGCTATGAC									
C45^a^	F: CATGAATGATTACGATTCGAGC	(AG)_20_	106–199	0.162	0.151	2	NS	0.281	60	JQ739901
	R:TGAGTGATTACTAGTGGAACCCA									
C76	F: AGGCGAAGCAAGAGGTTTTATCCAC	(CT)_20_	194–233	0.027	0.24	3	*	0.454	59	JQ739890
	R:GAAGAGGCAGAAAGAACTTACAGCG									
C106^a^	F:TTGCAGGTAGCAGGTTGT	(TCTA)_3_TCTG	383–431	0.029	0.585	5	*	0.656	57	JQ739891
	R:CAGACAGATAGATAGAGACGG	(TCTA)_3_TCAA(TCTA)_5_								
C115^a^	F: CGTAGTCTCTTTCGTCCTTTTCTGC	(CT)_17_	212–245	0.114	0.533	3	*	0.575	57	JQ739892
	R: TTACATGCCCTTTCCCTCGTTTG									
C117	F: GTACCTCCTTTTGAGTTTGTAAGG	(CT)_12_	150–173	0.028	0.406	3	*	0.55	55	JQ739893
	R: GTTCGTAAGCCTACTTTCTCA									
C119	F: CCACCATTGCTCTCAGTGTTA	(CT)_21_	251–292	0.139	0.45	3	*	0.458	57	JQ739894
	R: TAGATGTGTGTTTGTGTTCCG									
C130^a^	F: CCATTTCAAAGCAAACCCCTT	(CT)_3_CG(CT)_2_TG (CT)_19_	298–349	0.028	0.597	3	*	0.590	60	JQ739896
	R: TGACCCGCGATTATTTACCAC									
C132^a^	F: CAGTGGTTATTCTGGGGATT	(CT)_13_	477–502	0.118	0.674	5	*	0.687	58	JQ739897
	R: GGTTGTTTATGGCAGTAGCA									
C136^a^	F:GTAAAAGTCTCCTTCTACTTTCCCC	(CT)_22_	272–315	0.057	0.306	4	*	0.503	60	JQ739898
	R:CTCTCAAGCATGTTTGGATTGTAGC									
G10^a^	F: TCTTCTGTCCATCCCTCCATACT	(AG)_27_	220–273	0.088	0.327	3	*	0.444	60	JQ739899
	R: GATTGGTGGAAATCGACTTGTCT									

F: the forward primer sequences; R: the reverse primer sequences; *H*_O_: observed heterozygosity; *H*e: expected heterozygosity; *N*_a_: number of alleles; *D*: deviation from Hardy-Weinberg equilibrium; *T*_a_: annealing temperature; NS: not significant;

* : significant deviations from Hardy-Weinberg expectations after Bonferroni correction at α = 0.05.

The superscript ^a^ represents a primer pair that can be used for successful amplification in *Vigna unguiculata* (Linn.).
